# Robust Biomimetic Nacreous Aramid Nanofiber Composite Films with Ultrahigh Thermal Conductivity by Introducing Graphene Oxide and Edge-Hydroxylated Boron Nitride Nanosheet

**DOI:** 10.3390/nano11102544

**Published:** 2021-09-28

**Authors:** Cenkai Xu, Chengmei Wei, Qihan Li, Zihan Li, Zongxi Zhang, Junwen Ren

**Affiliations:** 1 College of Electrical Engineering, Sichuan University, Chengdu 610065, China; 2018141411155@stu.scu.edu.cn (C.X.); weichengmei@stu.scu.edu.cn (C.W.); 2020223035177@stu.scu.edu.cn (Z.L.); 2 College of Aviation Engineering, Civil Aviation Flight University of China, Guanghan 618307, China; 2018141441007@stu.scu.edu.cn; 3 Electric Power Research Institute, State Grid Corporation of Sichuan Province, Chengdu 610072, China; 2019223035137@stu.scu.edu.cn

**Keywords:** composite film, thermal conductivity, boron nitride, hydrogen bond, aramid nanofibers

## Abstract

Dielectric materials with excellent thermally conductive and mechanical properties can enable disruptive performance enhancement in the areas of advanced electronics and high-power devices. However, simultaneously achieving high thermal conductivity and mechanical strength for a single material remains a challenge. Herein, we report a new strategy for preparing mechanically strong and thermally conductive composite films by combining aramid nanofibers (ANFs) with graphene oxide (GO) and edge-hydroxylated boron nitride nanosheet (BNNS-OH) via a vacuum-assisted filtration and hot-pressing technique. The obtained ANF/GO/BNNS film exhibits an ultrahigh in-plane thermal conductivity of 33.4 Wm^−1^ K^−1^ at the loading of 10 wt.% GO and 50 wt.% BNNS-OH, which is 2080% higher than that of pure ANF film. The exceptional thermal conductivity results from the biomimetic nacreous “brick-and-mortar” layered structure of the composite film, in which favorable contacting and overlapping between the BNNS-OH and GO is generated, resulting in tightly packed thermal conduction networks. In addition, an outstanding tensile strength of 93.3 MPa is achieved for the composite film, owing to the special biomimetic nacreous structure as well as the strong π−π interactions and extensive hydrogen bonding between the GO and ANFs framework. Meanwhile, the obtained composite film displays excellent thermostability (*T*_d_ = 555 °C, *T*_g_ > 400 °C) and electrical insulation (4.2 × 10^14^ Ω·cm). We believe that these findings shed some light on the design and fabrication of multifunctional materials for thermal management applications.

## 1. Introduction

With the rapid development of advanced electronics to higher voltages, higher frequency, and higher temperature, efficient heat dissipation of devices has become a great challenge [1,2,3,4,5]. Advanced thermal management materials (TMMs) with high thermal conductivity and excellent mechanical properties offer significant promise in alleviating the issue of heat concentration. Polymeric TMMs have attracted tremendous attention, owing to their unique characteristics of easy processing, lightweight, and excellent flexibility [6,7]. However, the intrinsically low thermal conductivity and heat-resistance temperature of most of the polymers restricts their further applications. Recently, aramid nanofibers (ANFs), as a novel one-dimensional (1D) nanoscale building block formed by interacting with each other via strong and highly aligned hydrogen-bonded networks of poly(p-phenylene terephthalamide) (PPTA) chains [8], have exhibited a great potential in the fabrication of multifunctional materials due to their nanoscale morphology, high aspect ratio, excellent mechanical strength, and thermostability [9,10,11,12]. Nevertheless, ANF-based composite films exhibit poor heat-conducting characteristics [11]. Incorporating thermally conductive fillers (such as carbon nanotubes [13], graphene [14], silver particles and nanowires [15], aluminum oxide [16], aluminum nitride [17], silicon carbide [18], boron nitride [19,20], etc.) into the ANF matrix has been demonstrated to be an effective method to improve the thermal conductivity of the composite films. Zhang and coworkers reported that with a core-sheath graphene fiber wrapped by ANF, an ultimate tensile stress of 380 MPa was achieved for the hybrid fiber. Unfortunately, the high electric conductivity still prevents its further application in integrated circuits and high power devices [21]. Therefore, it is necessary to find thermally conductive yet electric insulating fillers that can effectively establish heat transport channels as well as act as electric barriers in the composites.

Boron nitride nanosheet (BNNS), as a structural analog of graphene (also called “white graphene” [22]), exhibits inherent high thermal conductivity (~2000 Wm^−1^ K^−1^) [23], wide band gap (~5.9 eV) [24], low dielectric constant (~3.9), high thermal stability [25], and large aspect ratio, and has been proved to be a good choice to set up a thermally conductive path in composites [26,27]. For instance, Xiao et al. prepared the ANF/boron nitride composite films with ultrahigh thermal conductivity of 122.5 Wm^−1^ K^−1^. However, the mechanical properties of the composite films decreased significantly to ~30 MPa, owing to the poor stress transfer between ANF framework and thick BN platelets [20]. Natural nacre, a binary composite system that contains 95 vol% two-dimensional (2D) inorganic platelets and 5 vol% organic polymers, evolves into a well-ordered ‘‘brick-and-mortar’’ architecture to provide extraordinary mechanical properties [28,29]. A nacre-like ANFs/montmorillonite nanocomposite film with layered microstructure was successfully constructed by Si et al. via simple vacuum-assisted filtration, and the “brick-and-mortar” structure of 2D platelets and polymer contributes to the excellent mechanical properties of composite film [30]. Lei et al. reported an ultrathin, highly robust, super-flexible, and thermostable composite film via engineering ANFs with Ti_3_C_2_T_x_ (MXene) into a hierarchical 2D/1D brick-and-mortar architecture, and achieved an unprecedented tensile strength (300.5 MPa) at 40 wt.% MXene loading [31].

In addition, although the heat conduction network of the polymer matrix can be well constructed while fillers form a random close-packed structure, the high fillers loading will generally deteriorate the superior mechanical performance of a polymer matrix [32]. To address this issue, a solution for the combined use of hybrid fillers is proposed [33,34,35,36,37]. Zhao et al. reported a new strategy to synergistically enhance the thermal conductivity of epoxy composites with 2D BNNS and 0D boron nitride microspheres (BNMSs). The result shows that the thermal conductivity of BNNSs/BNMSs/epoxy composite (1.148 Wm^−1^ K^−1^) with a filler loading of 30 wt.% is approximately 28.0 and 51.5% higher than that of BNNSs/epoxy and BNMSs/epoxy composites, respectively [38]. Jiang et al. reported that the thermal conductivity of the polystyrene (PS) composites reached a maximum and exhibited the highest thermal conductivity enhancement up to 20% while the mass ratio of graphene oxide/hydroxylated boron nitride (GO/BN-OH) was 7:3. This demonstrated that the thermal conductivity of the PS composites could demonstrate significantly improved benefit from the synergistic effect of GO and BN-OH [39]. Graphene possesses ultrahigh thermal conductivity and a large aspect ratio, which is the best choice for the fabrication of thermally conductive composites [40,41,42]. However, the high electrical conductivity of graphene restricts the application of TMMs. GO sheets, as inorganic heat-dissipating filler are as important as graphene; it is found that the oxygen-containing groups on the surface of GO not only substantially enlarged the interlayer spacing of GO sheets, but effectively improve the dispersibility in the polymer matrix [43,44]. Most importantly, the oxygen-containing groups are not conducive to the conduction of carriers. Wang et al. fabricated composite films by blending GO sheets in ANFs, and enhanced the mechanical stability of composite films through the π–π stacking interactions between the ANFs and GO sheets in this system [45]. Note that excessive GO sheets may greatly reduce the reliability of polymer composites as TMMs due to their inherent electrical conductivity [46].

In this study, the biomimetic nacreous aramid nanofiber-based composite films with outstanding mechanical properties and high thermal conductivity were fabricated by employing GO sheets and edge-hydroxylated BNNS (BNNS-OH). Special “brick-and-mortar” structures, as well as strong hydrogen-bonding, are generated between the functional groups of the GO, hydroxyl group of BNNS-OH, and the amide group of ANFs, which plays a significant role in improving the mechanical properties of ANF/GO/BNNS composite films. In addition, tightly packed thermal conduction networks are established in the biomimetic nacreous layered structure, contributing to high thermal conductivity of the composite films. Meanwhile, the low content of GO in the composite film remains low electron transport efficiency, resulting in high electrical insulating properties of composite films.

## 2. Experimental Section

### 2.1. Materials

Kevlar 29^®^ yarn was purchased from DuPont (DuPont, Wilmington, DE, USA). Hexagon boron nitride (h-BN, average size of 10 μm, 99.5% purity) was provided by Qinhuangdao ENO High-Tech Material Development, Qinghuangdao, China. GO (single-layer graphene oxide, >99% purity) was purchased from Gaoxi Technology Co., Ltd., Hangzhou, China. Potassium hydroxide (KOH), sodium hydroxide (NaOH), and lithium chloride (LiCl) were purchased from Aladdin Biochemical, Shanghai, China. Dimethyl sulfoxide (DMSO), isopropyl alcohol (IPA), and deionized water (H_2_O) were obtained from Chengdu Kelong Chemical Reagent Co., Ltd., Chengdu, China. All chemicals were analytical reagent grade and used without further purification.

### 2.2. Fabrication of ANFs, BNNS-OH, and ANF/GO/BNNS Composite Films

**Figure 1:**
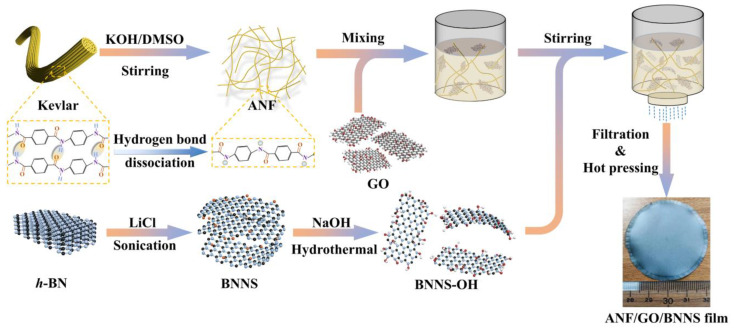
Schematic illustration for fabrication of the ANF/GO/BNNS composite film.

The overall fabrication schematic is illustrated in Figure 1. Kevlar 29^®^ yarn was cut short, put into a beaker, and ultrasonically washed for 48 h in an anhydrous ethanol bath, then the cleaned Kevlar 29^®^ yarn was dried in a vacuum oven at 45 °C. 1.6 g of the treated Kevlar 29^®^ yarn and 2.4 g KOH were added to the mixed solution of 12.8 mL H_2_O and 320 mL DMSO, and a dark red ANFs/ DMSO dispersion was obtained by stirring the mixture at 800 rpm for 1 week at 30 °C.

BNNS-OH was prepared by liquid-phase exfoliation and subsequent hydroxyl functionalization. First, 1 g of raw h-BN and 1 g of LiCl were added to a mixed solvent of 75 mL IPA and 25 mL H_2_O. The suspension was sonicated (500 W) in a bath for 6 h, and was then hydrothermally treated at 180 °C for 24 h in a Teflon-lined stainless-steel autoclave to enlarge the layer spacing of h-BN. Subsequently, BNNS was collected by centrifuging at 2000 rpm for 10 min. The obtained BNNS was re-dispersed in 200 mL of NaOH solution (5 M) by sonication and stirring for 3 h and was then hydrothermally treated again in a Teflon-lined stainless-steel autoclave at 180 °C for 24 h to functionalize the BNNS. The resulting suspension was then filtered and washed thoroughly with H_2_O to remove the excess lye and ions until the pH of the filtrate was 7. The obtained BNNS-OH was collected and dried in a vacuum oven at 60 °C for 48 h.

The ANF/GO/BNNS composite film was fabricated by simple vacuum-assisted filtration of the uniformly distributed ANFs, GO sheets, and BNNS-OH mixture dispersion, and followed by a hot-pressing process. Briefly, 10 mg GO sheets and 10 mg BNNS-OH were dispersed uniformly in DMSO solvent, respectively. 46 g DMSO solvent was added to 23 g ANFs/DMSO solution (100 mg ANFs) to dilution, and then 4% deionized water (2.76 g) was added to the diluted solution, then sonicated (500 W) in a bath for 1 h to partial protonation of ANFs. Then the GO/DMSO solution was added to the treated ANFs/DMSO solution and stirred magnetically at 800 rpm for 1 h at 25 °C, then the uniformly dispersed BNNS/DMSO solution was added, and magnetic stirring was continued at 25 °C for 2 h. The obtained ANF/GO/BNNS/DMSO dispersion was injected into 500 mL of H_2_O to form the colloidal ANF/GO/BNNS. Then, the colloidal ANF/GO/BNNS was separated by vacuum filtration using Buchner funnel to completely remove the residual DMSO and KOH until the filtrate is neutral. The purified colloidal ANF/GO/BNNS was added to 400 mL of H_2_O and violently cut at 14,000 rpm for 10 min to obtain a uniform ANF/GO/BNNS slurry. Then, the slurry was continuously vacuum-filtrated with a 0.2-µm-pore polytetrafluoroethylene (PTFE) membrane for over 8 h to obtain the ANF/GO/BNNS film, which was hot-pressed at 150 °C for 5 min and vacuum-dried at 45 °C for 48 h. Here the fabricated composite film was composed of 10 mg GO, 10 mg BNNS-OH, and 100 mg ANFs, and was labeled ANF/GO-10/BNNS-10. The preparation of other ANF/GO/BNNS composite films only needs to change the mass of BNNS and GO, and the steps remain the same. The ANF/GO-5/BNNS-X and ANF/GO-10/BNNS-X (X is 10, 20, 30, 40, 50) composite films were fabricated. The ANF film was prepared according to the same procedure of ANF/GO/BNNS composite films without GO sheets and BNNS-OH.

### 2.3. Characterization

The microstructure of Kevlar fibers and the cross-section of ANF film and ANF/GO/BNNS composite films were characterized by scanning electron microscopy (SEM, Quanta 250 FEG, FEI, Hillsboro, OR, USA,) under an acceleration voltage of 3.0 kV, and all samples were coated with a thin layer of gold to improve the conductivity of the samples. The morphologies of ANFs and BNNS-OH were observed by transmission electron microscope (TEM, JEM2100F, Tokushima, JEOL, Tokyo, Japan) at an accelerating voltage of 200 kV and atomic force microscopy (AFM, Bruker MultiMode 8, Bruker, Karlsruhe, Germany). X-ray diffraction (XRD, Philips, Amsterdam, The Netherlands) patterns were recorded on a Philips X’ Pert Pro MPD X-ray diffractometer operated with Cu-Kα radiation (λ = 0.154 nm) over the 2θ range of 15−60° with a scanning speed of 5°/min. The thermogravimetric analysis (TGA) of the samples was analyzed by TA Instruments high-resolution TGA 2950 thermogravimetric analyzer at a heating rate of 10 °C/min from 30 to 800 °C under the flow of N_2_ (20 mL/min). The chemical compositions and interactions of ANF/GO/BNNS composite films were tested using Fourier transform infrared spectrometer (FTIR, ThermoFisher, Shanghai, China) on a Nicolet 6700 spectrometer from 400 to 4000 cm^−1^. Differential scanning calorimetry (DSC) was performed on a TA 2100 calorimeter (PerkinElmer, Waltham, MA, USA) with a heating rate of 5 °C/min under an N_2_ flow (20 mL/min). The thermal diffusivities (α) of the composite films was investigated using a NETZSCH LFA 467 Laser Flash Apparatus (NETZSCH, Freistaat Bayern, Germany) at 25 °C, and the density (ρ) was calculated by ρ = *m*/*v*, where the *m* and *v* are the mass and volume of composite film, respectively. The *C*_p_ is the specific heat capacity of composite film and is measured using DSC with the Sapphire method. The mechanical properties of ANF film and ANF/GO/BNNS composite films were evaluated by a universal testing machine (Instron 5967, Canton, MA, USA) at a loading rate of 1 mm/min. Every sample was cut into strips with a length of 20 mm and a width of 5 mm for at least five tests to reduce the error of test results. To directly evaluate the heat dissipation capability of the composite films, a 3 W light-emitting diode (LED) chip was attached to the composite films with copper glue and connected to a direct-current power of 3 V, and the surface temperature of composite films was recorded by the infrared thermograph (FLIR T650sc, Washington, DC, USA). The electrical volume resistivity of composite films was tested on a Keithley 6517B resistivity test fixture (Tektronix, Wayne, NJ, USA).

## 3. Results and Discussions

**Figure 2:**
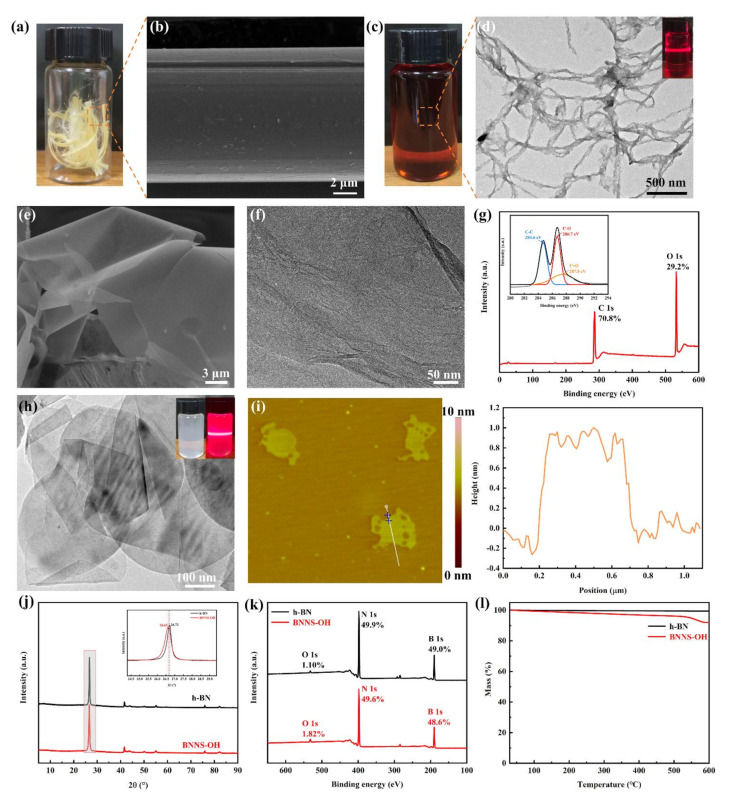
(**a**) Optical photograph of the macroscopic Kevlar fibers. (**b**) SEM image of Kevlar fibers. (**c**) Optical photograph of the ANFs dispersion. (**d**) TEM image of ANFs (inset: digital image of ANFs/DMSO solution with Tyndall effects). SEM (**e**) and TEM (**f**) images of GO. (**g**) XPS spectra of GO sheets (inset: XPS C1s spectra of GO). TEM (**h**) and AFM (**i**) images of BNNS-OH. Comparative (**j**) XRD patterns, (**k**) XPS profiles, and (**l**) TGA analysis between h-BN and BNNS-OH.

The macroscopic Kevlar fibers (Figure 2a) were approximately 11 μm in diameter, and a smooth surface lead to a poor interfacial adhesion, as shown in Figure 2b. The deterioration of composite properties is always related to the weak interfacial strength [9], therefore, it is particularly critical to improve the chemical inertness of the Kevlar fibers. A previous report has shown that Kevlar fibers that are exposed to a potassium hydroxide/dimethyl sulfoxide (KOH/DMSO) system abstracts the mobile protons from amide groups to generate the negatively charged nitrogen ions [47]. The electrostatic repulsion and the destroyed hydrogen bonding interactions between the polymer chains facilitate the transformation from Kevlar fibers to numerous ANFs. Meanwhile, physical tangles and π–π stacking in the polymer backbone hinder the further dissociation of ANFs into polymer chains [48].

Figure 2c shows the preparation of ANFs dissolved in DMSO, obtained a dark red, highly stable, and homogeneous ANFs/DMSO solution. The synthesized ANFs are ~50 nm in diameter and several micrometers in length, exhibiting a network-like structure (Figure 2d). The inset of Figure 2d shows the obtained ANFs/DMSO dispersion presented a strong Tyndall effect, indicating its colloidal characteristic and good dispersibility.

The GO exhibits a large surface and curly sheet with smooth surface morphology according to the results of SEM (Figure 2e). Meanwhile, the TEM image of GO (Figure 2f) also displays a single layer structure and slight wrinkles, indicating that the GO could be well utilized as an excellent support material with a large specific surface area [49]. XPS analysis was performed to characterize the chemical structure of GO. Figure 2g presents the XPS spectra of GO. The GO shows distinct peaks for C1s at 286.4 eV and O1s at 532.1 eV. The atomic fractions of carbon and oxygen are 70.8% and 29.2%, respectively [50]. For a detailed investigation, The C 1s spectrum of GO (inset of Figure 2g) exhibits the characteristic peaks of C−C at 284.6 eV, C−O at 286.7 eV, and C=O at 287.5 eV corresponding to the hydroxyl and carboxyl groups.

In Figure 2h, BNNS-OH remains with an intact crystalline structure and is highly electron transparent in TEM observations, indicating the prepared BNNS-OH possesses negligible defects and ultrathin nature after exfoliation and functionalization. The few-layer structures of BNNS-OH can also be clearly observed in the AFM image of BNNS-OH (Figure 2i), BNNS-OH with a lateral size of ~0.5 μm and an ultrathin thickness of ~1 nm is observed. It proves that the exfoliated BNNS-OH is composed of ~3 layers because an isolated BNNS is approximately 0.4–0.5 nm [7,51]. The decreased thickness and crystalline structures of BNNS-OH can be further confirmed from the X-ray diffraction (XRD) patterns analysis (Figure 2j). In comparison to h-BN, the typical (002) diffraction peak of BNNS-OH exhibits a tiny angle shift from 26.72° to 26.63°, suggesting the increased interplanar distance of BNNS-OH and the hexagonal lattices of the BNNS-OH are not damaged during the exfoliation and functionalization processes. In addition, the visible lower intensity and broader width of the (002) diffraction peaks of BNNS-OH than those of h-BN are observed, which further indicates the thickness of the BNNS-OH decreases [38]. The thinner thickness and well-retained crystalline structure of BNNS-OH are favorable for enhancing interfacial phonon coupling in composite films. The XPS profile of h-BN shows two strong boron and nitrogen peaks at ~190.1 and 398.1 eV, along with a small oxygen peak at ~532.1 eV. After exfoliation and functionalization, the intensity of the oxygen peak increased (from 1.10% for h-BN to 1.82% for BNNS-OH), indicating the formation of surface hydroxyl groups, which roughly corresponds to one hydroxyl group for every 67 B-N atoms (Figure 2k) [52]. Figure 2l demonstrates the TGA curves of h-BN and BNNS-OH. The weight loss of BNNS-OH increases with the increase in temperature and exhibits a weight loss of 8.1% at 600 °C, mainly due to the decomposition of the hydroxyl group grafted on the edge of BNNS. Besides, a slight loss of BNNS-OH was observed at temperatures below 150 °C, the loss originated from molecular water adsorption on the BNNS-OH surface [51]. In conclusion, the above characterizations and analysis confirm the successful fabrication of BNNS-OH with thin thickness and well-retained crystalline structure.

**Figure 3:**
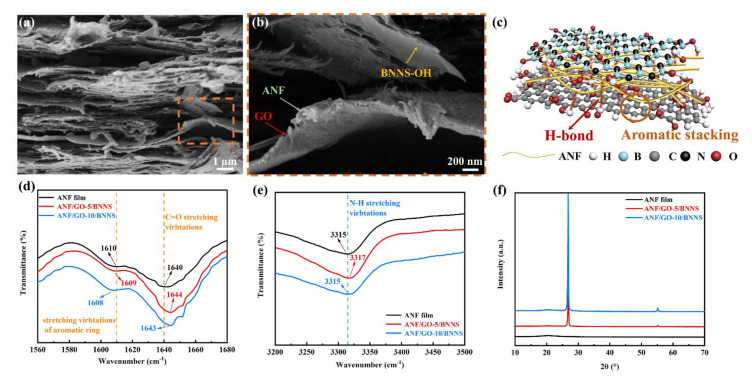
(**a**) Cross-section morphologies of ANF/GO/BNNS film at low magnification. (**b**) Partial enlarged view of cross-section morphologies of ANF/GO/BNNS film. (**c**) Diagram of hydrogen bonding and π–π stacking inner ANF/GO/BNNS composite film. (**d**,**e**) FT-IR spectra of ANF film, ANF/GO-5/BNNS, and ANF/GO-10/BNNS composite films. (**f**) XRD patterns of ANF film, ANF/GO-5/BNNS, and ANF/GO-10/BNNS composite films.

The ANF/GO/BNNS composite films were fabricated by vacuum-assisted filtration and hot-pressing methods to form a dense film. Figure 3a,b shows the cross-section morphologies; it is clearly observed that numerous ANFs are attached to the surface of these GO sheets and the surface of BNNS-OH is relatively smooth. This is because abundant oxygen-containing groups exist on the surface of GO to form strong hydrogen bonds with ANFs, and π−π interactions exist between the GO’s graphitic basal plane and the ANFs’ polymer backbone [53,54]. By contrast, the prepared BNNS-OH possess fewer hydroxyl groups, and the hydrogen bond between the ANFs and BNNS-OH is relatively weak. The interaction between ANFs, GO, and BNNS-OH is demonstrated in Figure 3c. Fourier transform infrared (FTIR) spectra was carried out to demonstrate these strong interfacial interactions among ANFs, GO, and BNNS-OH (Figure 3d,e). As can be seen, the FTIR spectrum of ANF film presents the band at 1640 cm^−1^ corresponding to the C=O stretching vibrations, the band at 1610 cm^−1^ corresponding to the stretching vibrations of aromatic ring, and the band at 3315 cm^−1^ corresponding to the N-H stretching vibrations [48]. The formation of new hydrogen bonds could be confirmed by the blue shift of C=O stretching vibrations to 1644 and 1643 cm^−1^ for the ANF/GO-5/BNNS and ANF/GO-10/BNNS composite films, respectively. Besides, the blue shift of N-H stretching vibrations to 3317 cm^−1^ for the ANF/GO-5/BNNS also indicates the formation of new hydrogen bonds between ANFs, GO sheets, and BNNS-OH. It is found that the strongest peak around 1610 cm^−1^ (the stretching vibrations of the aromatic ring of ANFs) underwent a gradual redshift upon the addition of more GO sheets. Such a result can be direct evidence for the existence of π–π interactions between ANFs and GO sheets [54,55]. The crystal structures are shown in X-ray diffraction (XRD) patterns; Figure 3f confirms the presence of among ANFs, GO, and BNNS-OH in the ANF/GO/BNNS composite films. The ANF film exhibited a strong peak at around 20.3° (d = 0.44 nm), assigned to a crystal plane of (110) [53]. The XRD patterns of the ANF/GO/BNNS composite films exhibited diffraction peaks of ANFs (~20.3°), GO (~26.8°), and BNNS-OH (~26.8°, ~42.9°, and ~55.2°). Although it is difficult to interpret because the diffraction peaks at ~26.8° are similar to that of both the GO sheets and the BNNS-OH, BNNS-OH also exhibits other diffraction peaks at ~42.9° and 55.2°, corresponding to the (100) and (101) crystal planes, respectively. Compared to ANF/GO-5/BNNS, the intensity of the diffraction peaks at ~26.8° of ANF/GO-10/BNNS tends to become stronger with the increasing of GO loading, which confirms the good crystal structure of GO sheets [24].

**Figure 4:**
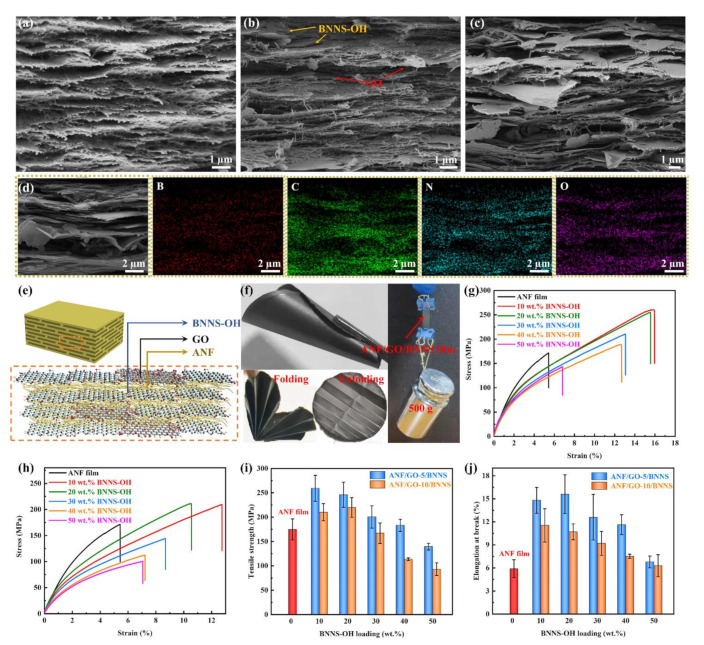
Cross-sectional SEM images of (**a**) ANF film, (**b**) ANF/GO-5/BNNS-10, (**c**) ANF/GO-10/BNNS-50 composite films. (**d**) Energy-dispersive X-ray mapping of elements B, C, N, and O in ANF/GO-10/BNNS-50 composite film. (**e**) Sketch of the nacre-mimetic layered structure. (**f**) Demonstration of high mechanical strength and origami foldability of ANF/GO/BNNS composite films. Typical stress−strain curves of (**g**) ANF/GO-5/BNNS and (**h**) ANF/GO-10/BNNS composite films. (**i**) Tensile strength and (**j**) elongation at break of the ANF/GO/BNNS composite films.

Figure 4a–c displays the SEM images for the cross-sectional morphologies of ANF film, ANF/GO-5/BNNS-10, and ANF/GO-10/BNNS-50 composite films. All the films exhibit a well-ordered layered structure, and the good dispersion of BNNS-OH and GO sheets in ANF/GO-5/BNNS-10 composite film. For ANF/GO-10/BNNS-50 composite film, with increasing content of GO sheets and BNNS-OH, the contact area between the high thermal conductivity fillers increases. In addition, the BNNS-OH and GO sheets are well distributed in the composite film, as can be confirmed from the energy dispersive X-ray mapping of elements B, C, N, and O in ANF/GO-10/BNNS-50 (Figure 4d). Moreover, the ANFs, GO sheets, and BNNS-OH are orderly arranged in the horizontal direction in the SEM images of ANF/GO/BNNS composite film, which is similar to the “brick-and-mortar” structure of the natural nacre. It is well observed that BNNS-OH nanosheets and GO sheets, which are regarded as the “brick” in the nacre-like structure, are integrated appropriately at the interfaces with the “mortar” (ANFs) (Figure 4e) and the “brick-and-mortar” layered structure always displays an excellent mechanical property [56]. As shown in Figure 4f, the ANF/GO/BNNS composite film can endure the bending, stretching of heavy weight (500 g), as well as complex folding without any breakages.

The typical stress–strain curves of ANF/BNNS composite films are shown in Figure S1. It can be observed that the tensile strength of ANF/BNNS composite films decreases significantly with the increase of BNNS contents. In addition, at the same filler contents, the tensile strength of ANF/GO composite film is much higher than those of ANF/BNNS composite film (Figure S2). Therefore, the GO was utilized to further enhance the thermal conductivity and mechanical properties of ANF/BNNS composite film. The mechanical properties of ANF/GO-5/BNNS and ANF/GO-10/BNNS composite films with different contents of BNNS-OH were examined by tensile testing as shown in Figure 4g,h, respectively.

Meanwhile, combining tensile strength, elongation at break of ANF/GO/BNNS composite films are displayed (Figure 4i,j). It can be easily observed that the mechanical properties of ANF/GO-5/BNNS are stronger than that of ANF/GO-10/BNNS because of the stronger hydrogen bond formed between GO sheets and ANFs, while the addition of 5 wt.% GO sheets in composite films and the conclusion is demonstrated in the FTIR of ANF/GO/BNNS. The tensile strength and elongation at break of the ANF/GO-5/BNNS-10 composite films were 259.4 MPa and 14.8%, which is 48.4% and 151% higher than ANF film’s tensile strength and elongation at break of 174.8 MPa and 5.9%, respectively. The enhanced mechanical properties of the ANF/GO-5/BNNS-10 composite films are mainly ascribed to the extensive hydrogen bonding and π−π interactions between the ANFs and the GO sheets, and a tightly stacked, orderly multilayered nacre-like structure among ANFs, GO sheets, and BNNS-OH sheets [31,53]. For the ANF/GO/BNNS with a certain amount of GO sheets, the mechanical properties of ANF/GO/BNNS decrease with an increasing amount of BNNS-OH to some degree. The reason is that the fewer hydroxyl groups grafted on the edge of BNNS-OH, and the hydrogen bond formed between BNNS-OH and ANFs is weaker. In addition, the excessive introduction of BNNS-OH will cause insufficient “mortar” between the connecting “bricks”, which introduces structural defects and stress concentration points to the composite film [57].

**Figure 5:**
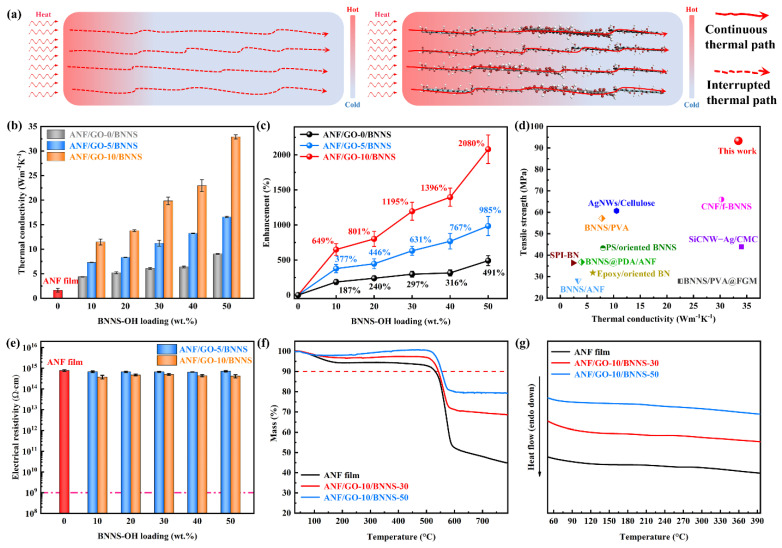
(**a**) Schematic showing potential thermal conduction mechanisms within the ANF film and ANF/GO/BNNS composite film. (**b**) In-plane thermal conductivity and (**c**) thermal conductivity enhancement of the composite films as a function of contents of GO and contents of BNNS-OH. (**d**) Comparison of thermal conductivity and tensile strength between the ANF/GO/BNNS composite film with other composites films reported in the literature. (**e**) Volume resistivity, (**f**) TGA curves, and (**g**) DSC curves of ANF film, ANF/GO-10/BNNS-30, and ANF/GO-10/BNNS-50 composite films.

Figure 5a shows the schematic illustrating the potential thermal conduction mechanisms within the ANF film and ANF/GO/BNNS composite film. When heat is applied from one end of the ANF film and ANF/GO/BNNS composite film, the thermal conductivity of the ANF/GO/BNNS composite film is significantly improved because multiple thermal transport pathways are possible with GO/BNNS-OH and its oriented lamellae forming a continuous network structure as can see from Figure 4c. Figure 5b shows the in-plane thermal conductivities (*λ*) of the ANF/BNNS, ANF/GO-5/BNNS, and ANF/GO-10/BNNS thermally conductive composite films, respectively. The values of *λ* for the composite films were calculated from Equation (1) using the measurement results of *C*_p_, *α*, and *ρ* [27]:

*λ* = *C*_p_ × *α* × *ρ* (1)

To further illustrate the effectiveness of improving the thermal conductivity, a parameter *η* was introduced, which is defined as follows [58]:

*η* = (*λ_c_* − *λ_m_*)/*λ_m_* × 100% (2)

where *λ_c_* and *λ_m_* represent the thermal conductivity of the composite films and pure ANF films, respectively. The *η* values are shown in Figure 5c. As can be seen, with the increase of BNNS-OH loading, the λ of the ANF/GO/BNNS composite films show a dramatic increase and achieve an ultrahigh value of 33.4 Wm^−1^ K^−1^ for the ANF/GO-10/BNNS-50 composite film, which is 2080% higher than that of the ANF film (~1.55 Wm^−1^ K^−1^). For ANF/GO-10/BNNS-50 composite films, ANFs, GO sheets, and BNNS-OH tend to be oriented horizontally via vacuum-assisted filtration and hot-pressing, and the large GO sheets can intercalate the gap between BNNS-OH and bridge the separated BNNS-OH, contribute to the formation of the more effective thermal conductive networks. Besides, the strong hydrogen bonds interaction of BNNS-OH, GO sheets, and ANFs, as well as π–π conjugation interactions between ANFs and GO sheets facilitate the reduction of the thermal interface resistance in the ANF/GO/BNNS composite films. To visualize the mechanical and thermal conductivity properties together, we plot the tensile strength vs thermal conductivity of our results compared against other composite films collected from the literature (Figure 5d and Table 1). The plot shows that the ANF/GO/BNNS composite films here exhibit a good combination of mechanical properties and superior in-plane thermal conductivity performances as compared to most other composite films, highlighting its superiority in the development of high-performance TMMs.

Figure 5e displays the electrical resistivity of the ANF film, ANF/GO-5/BNNS, and ANF/GO-10/BNNS composite films as a function of BNNS-OH loading. The ANF film shows a high electrical resistivity of 7.8 × 10^14^ Ω·cm, and exceeds the standard of electrical insulation (10^9^ Ω·cm). The addition of GO sheets and BNNS-OH leads to a negligible decrease of electrical resistivity owing to the wide band-gap of BNNS-OH with ultrahigh electrical resistivity as an electron transmission barrier in the ANF/GO/BNNS composite film, and the contents of GO sheets are too low to form an effective electron channel.

In addition, the thermal stabilities of the ANF/GO/BNNS composite films were verified by the TGA results (Figure 5f). As can be seen, the degradation rate decreased with an increase of BNNS-OH mass percent due to the high thermal stability of BNNS-OH. ANF film’s decomposition temperature of 10 wt.% weight loss (*T*_d_) was 530 °C, owing to the high thermal durability of the ANFs, the *T*_d_ of ANF/GO-10/BNNS-30, and ANF/GO-10/BNNS-50 increases by 13 and 25 °C, respectively. The reason can be ascribed to the high thermal durability and high intrinsic heat capacity of the GO sheets and BNNS-OH, as well as the “tortuous path effect” caused by the “brick-and-mortar” structure. Meanwhile, DSC results also show thermal stabilities of ANF film and ANF/GO/BNNS composite films (Figure 5g). The glass transition temperatures of all the films are higher than 400 °C due to PPTA polymer chains with sufficient amide bonds and strong interaction between the ANFs, GO, and BNNS-OH, indicating an excellent potential for electronic applications at high temperature.

**Figure 6:**
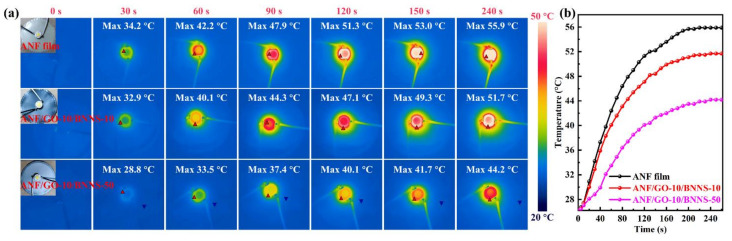
(**a**) Thermal infrared imaging of the LED chip at different working times using various heat spreaders of the ANF film, ANF/GO-10/BNNS-10, and ANF/GO-10/BNNS-50 (inset: Photographs of ANF film-based, ANF/GO-10/BNNS-10 composite film-based, and ANF/GO-10/BNNS-50 composite film-based LED devices). (**b**) Corresponding temperature-time curves.

To evaluate the cooling efficiency of this film in real operating conditions, the pure ANF film, ANF/GO-10/BNNS-10, and ANF/GO-10/BNNS-50 were used, respectively, for heat dissipation of high-power LED modules (3 W, see Figure 6a) with an ambient temperature of 20 °C. Note that the lifetime of LED chip is closely related to the operating temperature, suggesting that every 10 °C rise of temperature can lead to a decrease by half in its lifetime [67]. As expected, a lower hotspot temperature is displayed in the ANF/GO-10/BNNS-50 composite films as heat spreaders with respect to that of the ANF film and ANF/GO-10/BNNS-10 composite film substrate, indicating that the higher thermal conductivity of ANF/GO-10/BNNS-50 composite film provides an effective heat transfer rate. Figure 6b shows the corresponding temperature evolution of the ANF film and ANF/GO-10/BNNS composite films with 30 wt.% and 50 wt.% BNNS-OH loading, respectively. The ANF/GO-10/BNNS-50 composite film exhibits the lowest hotspot temperature, demonstrating the high efficiency of heat transfer. In short, the ANF/GO-10/BNNS-50 composite films with excellent mechanical properties, superior in-plane thermal conductivity performances, high electrical resistivity, and thermal durability would contribute to their applications in the thermal management of advanced electronics.

## 4. Conclusions

In summary, this work demonstrates the fabrication of ultrahigh thermally conductive and mechanically strong ANF/GO/BNNS composite films with a “brick-and-mortar” structure for high-performance thermal management materials via the vacuum-assisted filtration followed by a hot-pressing approach. The microstructures, thermal conductivity, mechanical properties, electrical insulation, and thermal stability of the nacre-like composite films are investigated in detail. The ANF/GO/BNNS composite film with GO content of 10 wt.% and BNNS-OH content of 50 wt.% shows outstanding mechanical properties with a tensile strength of 93.3 MPa, benefiting from the high-performance ANF substrate, the π−π interactions, and extensive hydrogen-bonding interaction, as well as the “brick-and-mortar” structure. Meanwhile, the ANF/GO-10/BNNS-50 composite film exhibits an exceptional thermal conductivity of 33.4 Wm^−1^ K^−1^ due to highly effective thermally conductive networks are constructed by the addition of GO sheets with high aspect ratio and high contents of BNNS-OH, and the good interface compatibility between ANFs, GO, BNNS-OH further reduces interface thermal resistance. Furthermore, high electrical insulation and remarkable thermal stability are simultaneously achieved for the ANF/GO/BNNS. The results demonstrate that ANF/GO/BNNS composite film with superior thermal management performance has great potential for application in modern integrated electronics and high-power electrical devices. More importantly, the combination of excellent mechanical, insulating, and thermally conductive properties for ANFs composite films open up opportunities to design advanced insulation system for generators, motors, transformers, and wide bandgap semiconductors.

## Figures and Tables

**Table 1:** Comparison of in-plane thermal conductivity and tensile strength of ANF/GO/BNNS composite film with other composite films reported in the literature.

Composite Films	Thermal Conductivity (Wm^−1^ K^−1^)	Tensile Strength (MPa)	References
BNNS/ANFs	3.33	28.2	[57]
BNNS@PDA/ANFs	3.94	36.8	[57]
SPI-BN	2.40	36.4	[59]
Epoxy resin/oriented BN	6.09	31.8	[60]
BNNS/PVA	7.80	57.2	[61]
PS/oriented BNNS	8.00	43.3	[62]
AgNWs/cellulose	10.55	60.7	[63]
BNNs/PVA@FGM	22.5	27.9	[64]
CNF/f-BNNS	30.3	66.0	[65]
SiC NW−Ag/CMC	34.0	43.9	[66]
ANF/GO/BNNS	33.4	93.3	This work

## Data Availability

The data presented in this study are available upon request from the corresponding author.

## Supplementary Materials

The following are available online at https://www.mdpi.com/article/10.3390/nano11102544/s1. Figure S1: Typical stress-strain curves of ANF/BNNS-OH films. Figure S2: Typical stress-strain curves and tensile strength of ANF/GO-5 and ANF/GO-10 films.
