# Supplementary material for: Robust Biomimetic Nacreous Aramid Nanofiber Composite Films with Ultrahigh Thermal Conductivity by Introducing Graphene Oxide and Edge-Hydroxylated Boron Nitride Nanosheet
Source: Nanomaterials (Basel). 2021 Sep 28;11(10):2544. doi: 10.3390/nano11102544 (PMC8539025; doi:10.3390/nano11102544)
Supplement: Supplementary file 1 [file nanomaterials-11-02544-s001.zip › nanomaterials-1353584-supplementary.pdf]

Supplementary Mateials

Robust Biomimetic Nacreous Aramid Nanofiber Composite Films with Ultrahigh Thermal Conductivity by Introducing Graphene Oxide and Edge-Hydroxylated Boron Nitride Nanosheet

Cenkai Xu [1], Chengmei Wei [1], Qihan Li [2], Zihan Li [1], Zongxi Zhang [3] and Junwen Ren [1,*]

[1] College of Electrical Engineering, Sichuan University, Chengdu 610065, China; 2018141411155@stu.scu.edu.cn (C.X.); weichengmei@stu.scu.edu.cn (C.W.); 2020223035177stu.scu.edu.cn (Z.L.)
[2] College of Aviation Engineering, Civil Aviation Flight University of China, Guanghan 618307, China; 2018141441007@stu.scu.edu.cn
[3] Electric Power Research Institute , State Grid Corporation of Sichuan Province, Chengdu 610072, China; 2019223035137@stu.scu.edu.cn
* Correspondence: myboyryl@scu.edu.cn

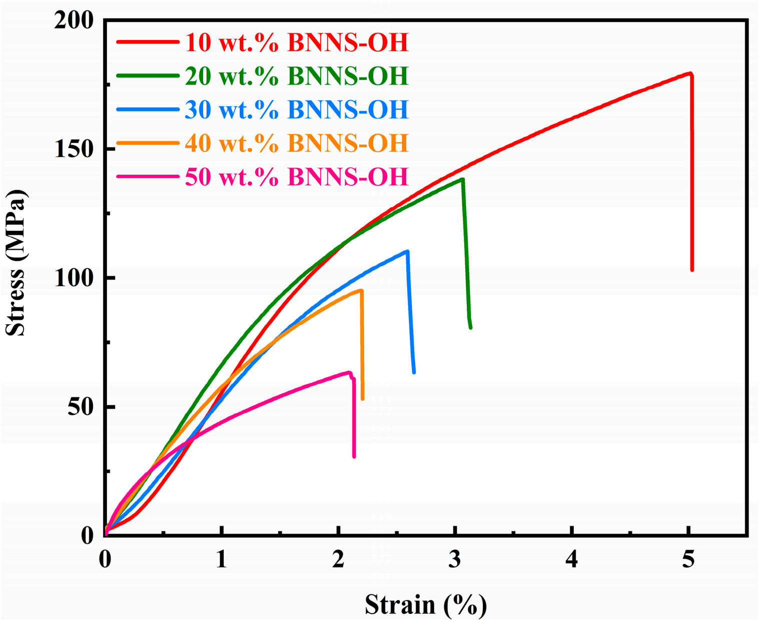

Figure S1. Typical stress-strain curves of ANF/BNNS-OH films.

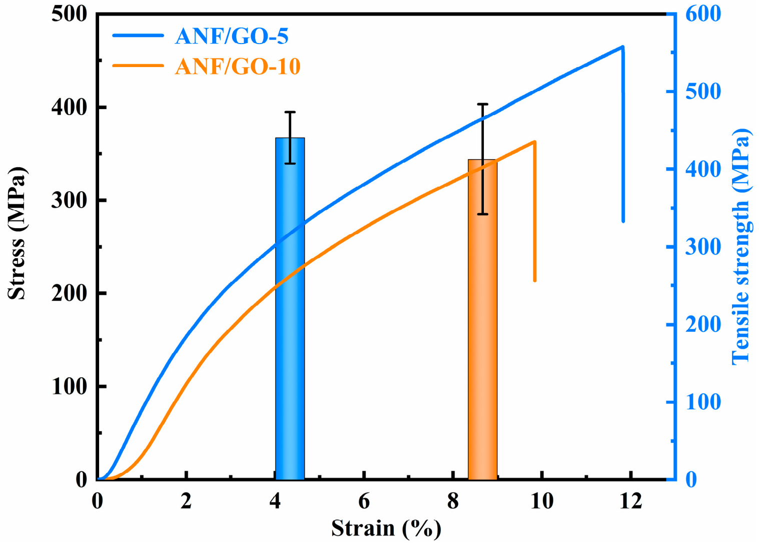

Figure S2. Typical stress-strain curves and tensile strength of ANF/GO-5 and ANF/GO-10 films.
